# Neurosteroids and their potential as a safer class of general anesthetics

**DOI:** 10.1007/s00540-023-03291-4

**Published:** 2024-01-22

**Authors:** Hiroki Tateiwa, Alex S. Evers

**Affiliations:** 1 Department of Anesthesiology and Intensive Care Medicine, Kochi Medical School, Kochi, Japan; 2 Department of Anesthesiology, Washington University School of Medicine, 660 S Euclid Ave, St. Louis, MO 63110 USA

**Keywords:** Neurosteroids, GABA_A_ receptor, Alphaxalone, Allopregnanolone

## Abstract

Neurosteroids (NS) are a class of steroids that are synthesized within the central nervous system (CNS). Various NS can either enhance or inhibit CNS excitability and they play important biological roles in brain development, brain function and as mediators of mood. One class of NS, 3α-hydroxy-pregnane steroids such as allopregnanolone (AlloP) or pregnanolone (Preg), inhibits neuronal excitability; these endogenous NS and their analogues have been therapeutically applied as anti-depressants, anti-epileptics and general anesthetics. While NS have many favorable properties as anesthetics (e.g. rapid onset, rapid recovery, minimal cardiorespiratory depression, neuroprotection), they are not currently in clinical use, largely due to problems with formulation. Recent advances in understanding NS mechanisms of action and improved formulations have rekindled interest in development of NS as sedatives and anesthetics. In this review, the synthesis of NS, and their mechanism of action will be reviewed with specific emphasis on their binding sites and actions on γ-aminobutyric acid type A (GABA_A_) receptors. The potential advantages of NS analogues as sedative and anesthetic agents will be discussed.

## Introduction

In 1941, Hans Selye demonstrated that intraperitoneal administration of selected pregnane and androstane steroids produced sedation and anesthesia in rats [1]. About 40 years later, in the 1980s, investigations by Baulieu and colleagues demonstrated that bioactive steroids similar to those that produced anesthesia were synthesized from cholesterol in the CNS, and termed these endogenous steroids as “neurosteroids” [2]. Subsequently, NS were suggested to play important roles in both nervous system development and behavioral modulation [3]. The finding that endogenously produced steroids could produce anesthesia sparked interest in both the endogenous role of these steroids in behavior and their therapeutic potential as anesthetic agents. An active search for a clinically applicable steroid anesthetic (see “Neurosteroids as anesthetics”) resulted in the introduction of several NS analogue anesthetics into clinical practice, culminating with Althesin (Glaxo, London, UK), which was widely used as an intravenous anesthetic in the 1970’s. Althesin was ultimately removed from clinical practice because of toxicity resulting from the excipient used in its formulation, and while it is still used as a veterinary anesthetic, it has not been replaced with a newer formulation or analogue.

**Fig. 1:**
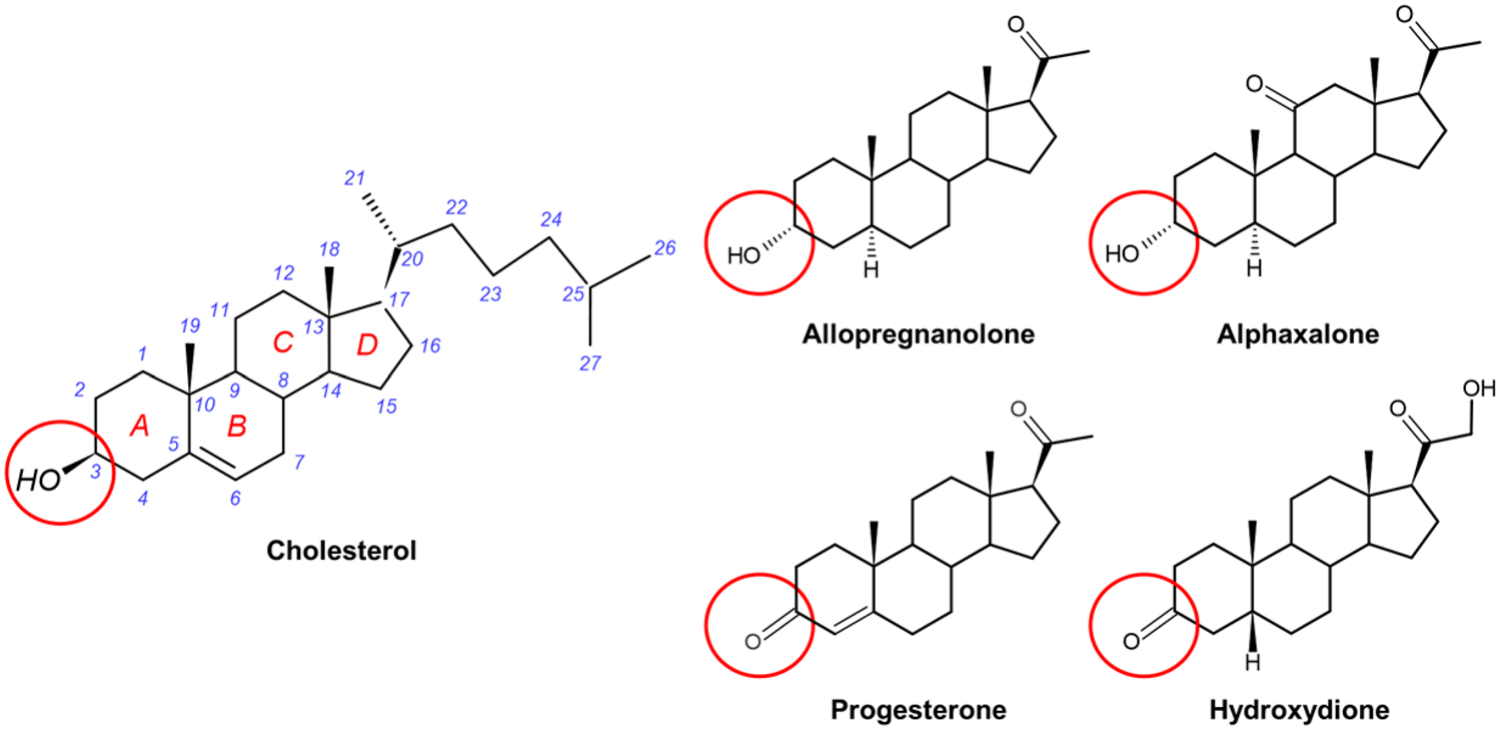
Chemical﻿ structure of cholesterol, allopregnanolone, progesterone and the synthetic anesthetic neurosteroids (alphaxalone and hydroxydione). Cholesterol has 27 carbons with a hydrocarbon (isooctyl) tail, a central steroid nucleus composed of four hydrocarbon rings (A, B, C, and D), and a hydroxyl group. The numbering of the carbon atoms indicates their position on the steroid ring or hydrocarbon tail. C3 is a carbon located on the A ring (*circled*), and 3-OH indicates that the hydroxyl group is connected to C3. The stereochemical configuration of the groups at the 3, 17, 18, 19, and 21 positions are shown by a dashed wedge (α-configuration) or a solid wedge (β-configuration). Note that cholesterol has a 3β-OH, whereas allopregnanolone and alphaxalone have 3α-OH groups. Progesterone and hydroxydione have ketone groups at C3

While anesthetic NS were withdrawn from clinical use, research over the ensuing decades has illuminated some of the targets and mechanisms of NS action and introduced new analogues and formulations. This work has led to the clinical introduction of NS anti-depressants and anti-epileptics, to exploration of their use as cognitive enhancers and therapeutics for neurodegenerative disorders (Table 1) and to renewed interest in NS as general anesthetics. Brexanolone, a new formulation of the NS allopregnanolone (AlloP (Fig. 1)), has demonstrated efficacy for treatment of postpartum depression (PPD) [4, 5] and was the first drug approved by the FDA for treatment of PPD (2019). Ganaxolone, a 3β-methylated analogue of AlloP, was approved by the FDA for treatment of seizures associated with CDKL5 deficiency disorder in 2022, making it the first NS to be approved for treatment of epilepsy [6]. However, NS have not been clinically reintroduced as anesthetics, in part because propofol served as an adequate replacement. NS do have potential advantages over current clinical anesthetics, notably their high therapeutic index (safety) and neuroprotective effects [7–9]. This review serves as an introduction to the NS field, describing the pathways of NS biosynthesis, their molecular targets and known binding sites and analyzing the historical experience with clinical use of NS as anesthetics with an emphasis on their development as anesthetics with significant advantages over currently available agents.

**Table 1:** Therapeutic targets of neurosteroids

Therapeutic target	Neurosteroids	References
Anxiety and depression	AlloP, Brexanolone	[4, 5, 139, 140]
Sedation/Anesthesia	AlloP, Progesterone, Alphaxalone	[1, 7–9, 45, 118, 141]
Epilepsy	AlloP, THDOC, Ganaxolone	[6, 61, 142, 143]
*Neurodegenerative disorder*		
Alzheimer’s disease	AlloP, PS, DHEAS	[144–148]
Parkinson’s disease	AlloP	[149–151]
Multiple sclerosis	AlloP, DHEA, 17β-estradiol, estriol	[152–155]
Memory enhancement	PS, DHEAS	[156–158]
Neuropathic pain	AlloP	[159–161]

## Biosynthesis of neurosteroids

Steroids such as pregnenolone, dehydroepiandrosterone (DHEA), and their sulfated metabolites are present in higher concentrations in rat brain than in plasma [10, 11]. These steroids persist in the nervous system after gonadectomy and/or adrenalectomy, indicating that they are synthesized in the brain [12]. The endogenous synthesis of steroids in the nervous system has been confirmed in human brains [13, 14]. Steroids primarily synthesized by central nervous system tissue are referred to as “neurosteroids” to differentiate them from steroids derived from classical steroidogenic organs such as gonads, adrenals, and placenta [15].

**Fig. 2:**
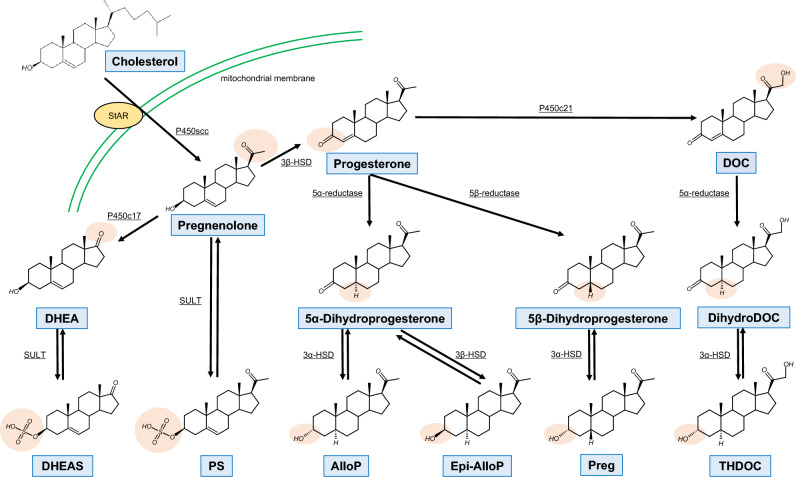
Biosynthetic pathways of neurosteroids. Abbreviations: 3α-HSD: 3α hydroxysteroid dehydrogenase, 3β-HSD: 3β hydroxysteroid dehydrogenase, *AlloP*: allopregnanolone, *DHEA*: dehydroepiandrosterone, *DHEAS*: dehydroepiandrosterone sulfate, *DOC*: deoxycorticosterone, *Epi-AlloP*: epi-allopregnanolone, *P450scc*: P450 side-chain cleavage, *Preg*: pregnanolone, *PS*: pregnenolone sulfate, *StAR*: steroidogenic acute regulatory protein, *SULT*: sulfotransferase, *THDOC*: tetrahydrodeoxycorticosterone

All steroids, including NS, are produced from cholesterol via several steps (Fig. 2). Cholesterol is a 27-carbon compound with a central polycyclic sterol structure composed of four hydrocarbon rings (A, B, C and D), a hydroxyl group attached at the 3-carbon on the A-ring and an 8-carbon isooctyl tail attached at the 17-carbon on the D-ring (Fig. 1). The first step of steroidogenesis begins with the transport of cholesterol into the mitochondria [16, 17]. Mitochondria are the critical site of neurosteroidogenesis, which involves multiple cytochrome P450 enzymes [18]. In this first step, the steroidogenic acute regulatory protein (StAR) on the outer mitochondrial membrane (OMM) plays an important role in transporting cholesterol into the mitochondrion [17, 19]. Other cholesterol transport proteins, such as translocator protein (TSPO) [20, 21] may also contribute to cholesterol transport across the OMM. A second step occurs on the inner mitochondrial membrane, where the isooctyl tail of cholesterol is cleaved to produce pregnenolone (3β-hydroxypregn-5-en-20-one). This reaction is catalyzed by P450 side-chain cleavage (P450scc) via a series of three distinct chemical reactions: 20α-hydroxylation, 22-hydroxylation, and scission of the C20–C22 carbon bond [22]. Homozygous deletion of the gene for P450scc eliminates all steroidogenesis [23, 24], confirming the critical role of this single enzyme [25].

Pregnenolone can be converted either to progesterone (Figs. 1 and 2) by the enzyme 3β-hydroxysteroid dehydrogenase (3β-HSD), or to DHEA through a series of reactions catalyzed by P450c17. Progesterone crosses into the cytosol via passive diffusion across the OMM [17]. From there, it can be either secreted as a hormone (e.g. in the ovaries) or further metabolized into pregnane NS, such as 3α-hydroxy-5α-pregnan-20-one (AlloP), 3α-hydroxy-5β-pregnan-20-one (Preg) and 3α,21-dihydroxy-5β-pregnan-20-one (THDOC). Specifically, progesterone is converted to 5α-dihydroprogesterone or 5β-dihydroprogesterone by the enzymes steroid 5α-reductase or 5β-reductase, respectively. Subsequently, 5α-dihydroprogesterone is converted to AlloP by 3α-hydroxysteroid dehydrogenase (3α-HSD) or epiallopregnanolone (Epi-AlloP, 3β-hydroxy-5β-pregnan-20-one) by 3β-HSD. Similarly, 5β-dihydroprogesterone is transformed to Preg by 3α-HSD. Progesterone can also be hydroxylated at C21 by P450c21 to yield 11-deoxycorticosterone (DOC), followed by conversion to THDOC by 3α-HSD. A critical point in understanding NS pharmacology is that the 3α- and 3β-HSD reactions are reversible, so that exogenously applied 3α and 3β-OH steroids can be interconverted in vivo.

Pregnenolone and DHEA can also be converted to pregnenolone sulfate (PS) and dehydroepiandrosterone sulfate (DHEAS) by cytosolic sulfotransferase enzymes (SULT). The sulfation reactions are reversible, and steroid sulfates can be desulfated by steroid sulfatase. In addition to their synthesis in the CNS, some steroid precursors, such as deoxycorticosterone, are synthesized in the gonads, adrenal gland, and fetoplacental unit. These steroid precursors synthesized in the periphery can cross the blood–brain barrier due to their lipophilic structures and be transformed to NS.

AlloP, Preg, and THDOC are positive allosteric modulatory neurosteroids (PAM-NS) of GABA_A_ receptors, potentiating the GABA-induced gating of chloride currents [26–29]. In contrast, Epi-AlloP, PS and DHEAS are negative allosteric modulatory neurosteroids (NAM-NS) of GABA_A_ receptors and inhibit GABA-gated currents [30–32]. Details are discussed later.

## Neurosteroids as anesthetics

### (1) Development of neurosteroid anesthetics

Selye demonstrated that progesterone and DOC produce anesthesia in rodents. The anesthetic effect of these steroids was greater in female than in male animals and the onset of anesthesia was slow [1]. Both of these points suggest that the active anesthetic molecule is a metabolite of the administered steroids. While the active metabolite was not yet identified and the molecular target of action was unknown, substantial effort was directed at identifying a safe and efficacious anesthetic steroid for clinical usage. The first challenge was to identify steroid anesthetics that were sufficiently soluble for parenteral use. In 1955, P’an reported that hydroxydione (21-hydroxy-5beta-pregnane-3,20-dione) (Fig. 1), a water-soluble ester of pregnanedione, was an effective anesthetic. While the onset of anesthesia was delayed in comparison to thiopental sodium, it had a higher therapeutic index (LD_50_/ED_50_) and was introduced as an anesthetic for humans [33]. Undesirable side effects including pain on injection, prolonged onset, slow recovery and thrombophlebitis led to the gradual disappearance of hydroxydione from clinical practice [34].

Subsequent research led to the introduction of alphaxalone (3α-hydroxy-5α-pregnane-11,20 dione) (Fig. 1) as an intravenous anesthetic in the 1970s. Alphaxalone is a 3α-OH analogue of progesterone that is devoid of progestational, estrogenic, glucocorticoid, mineralocorticoid and thermolytic activity [35]. Unlike hydroxydione, it produces rapid onset of anesthesia. This is consistent with the demonstration that 3α-OH steroids are the active anesthetic compounds [36, 37] and that the slow anesthetic onset of 3-keto steroids such as hydroxydione, progesterone or pregnanedione is a consequence of their metabolism to the active 3α-hydroxy derivative. For example, metabolism of progesterone to the 3α-OH PAM-NS, AlloP, (see “Biosynthesis of neurosteroids”) accounts for the delayed onset of anesthesia observed in Selye’s original experiments. This may also explain the preferential anesthetic effect of progesterone in female animals since females have higher levels of the critical enzyme 3α-HSD [38]. Alphaxalone was marketed as Althesin™ and was extensively used as a clinical anesthetic from 1972 to 1984. Alphaxalone has poor water solubility and was formulated as a mixture of alphaxalone and its 21-acetoxy derivative, alfadolone acetate, dissolved in a 20% solution of polyoxymethylene castor oil surfactant (Cremophor™ EL, BASF, Ludwigshafen, Germany). Althesin was a preferred anesthetic agent because of its rapid onset and offset of action, absence of irritating effects on blood vessels, and minor cardiovascular and respiratory effects [34, 39]. However, it was ultimately withdrawn from clinical use because Cremophor produced infrequent but serious anaphylactoid reactions [40]. Minaxolone was subsequently developed as a water-soluble NS anesthetic [41]. While it produced rapid onset and recovery from anesthesia and less depression of the cardio-respiratory system than Althesin, minaxolone was never used clinically, due to the finding of possible carcinogenicity in rats.

### (2) A new neurosteroid anesthetic: Phaxan

The clinical experience with hydroxydione and Althesin highlight both the advantages and disadvantages of NS anesthetics. A major limitation of earlier NS anesthetics was formulation. Phaxan is a new formulation of alphaxalone using a cyclodextrin excipient (13% 7-sulfobutylether-β-cyclodextrin; Captisol™), rather than Cremophor. A study comparing Phaxan, Althesin, and propofol in rats demonstrated that Phaxan caused anesthesia with faster onset and recovery than propofol and had a therapeutic index significantly greater than either Althesin or propofol [7]. Phaxan also produced significantly less cardiovascular depression than propofol. These data indicate that a cyclodextrin formulation avoids the toxicity of Cremophor, and provides enhanced safety, perhaps by limiting the maximal free concentration of drug in plasma.

Phaxan has also been tested in humans. A randomized, double-blind, phase 1c human study showed similar recovery times, but significantly less cardiovascular depression with Phaxan than propofol. Furthermore, airway obstruction requiring intervention was observed in 9 of 12 subjects in the propofol group, but none in the Phaxan group. The mechanisms by which Phaxan and propofol produce significant differences in the cardiovascular and respiratory systems at equal hypnotic dose may involve different off-target actions of the two drugs. Alternatively, Phaxan and propofol might interact with different GABA_A_ receptor subunit combinations or proteoforms.

## The functional effect of neurosteroids on the GABA_A_ receptors

While NS were used as clinical anesthetics from 1955 to 1984, their pharmacological mechanism of anesthetic action was unknown. In the early 1980s, Harrison and Simmonds showed that alphaxalone and pentobarbitone both produce enhancement of GABA_A_ receptor currents [42], which is thought to be their mechanism of anesthetic action. NS have subsequently been shown to have specific interactions with a variety of proteins including GABA_A_ receptors, glycine receptors, tubulin, voltage-dependent anion channel-1 and -2 (VDAC), liver X receptors (LXR), pregnane X receptors (PXR) and T-type Ca^2+^ channels. It has recently been suggested that 3β-OH NS may be anesthetics that act via T-type Ca^2+^ channels, rather than GABA_A_ receptors [43, 44]. The effect of these agents is slow in onset and is more pronounced in female animals, reminiscent of the earlier work by Selye. It has subsequently been shown that the 3β-OH NS anesthetics are metabolized peripherally to 3α-OH NS which can act as GABA-ergic anesthetics [44]. Genetic knockout of T-type Ca2^+^ channels does produce some shift in NS anesthetic response, suggesting a possible minor contribution of these channels to NS anesthesia.

Additional evidence that GABA_A_ receptors are a key target of NS anesthesia arises from their enantioselective actions. Wittmer et. al. showed that the 3α-OH, 5α-reduced NS, AlloP, potentiates GABA_A_ currents and induces anesthesia in tadpoles and mice, whereas its enantiomer (ent-AlloP) has minimal effect on GABA_A_ currents and does not produce anesthesia [45]. These data show that the actions of NS as GABA_A_ receptor modulators and as anesthetics are correlated. Enantiomers of hydrophobic ligands have equal solubility in lipids and are equally effective at disrupting lipid bilayers. Thus, the demonstration of AlloP enantioselectivity also provides evidence that NS actions result from binding to specific protein sites rather than from a nonspecific interaction with the lipid bilayer. GABA_A_ receptors are now thought to be the major target for NS anesthetic action. As background for a review of NS actions on GABA_A_ receptors, we first provide a brief summary of GABA_A_ receptor structure and function:

### (1) Overview of GABA_A_ receptors

**Fig. 3:**
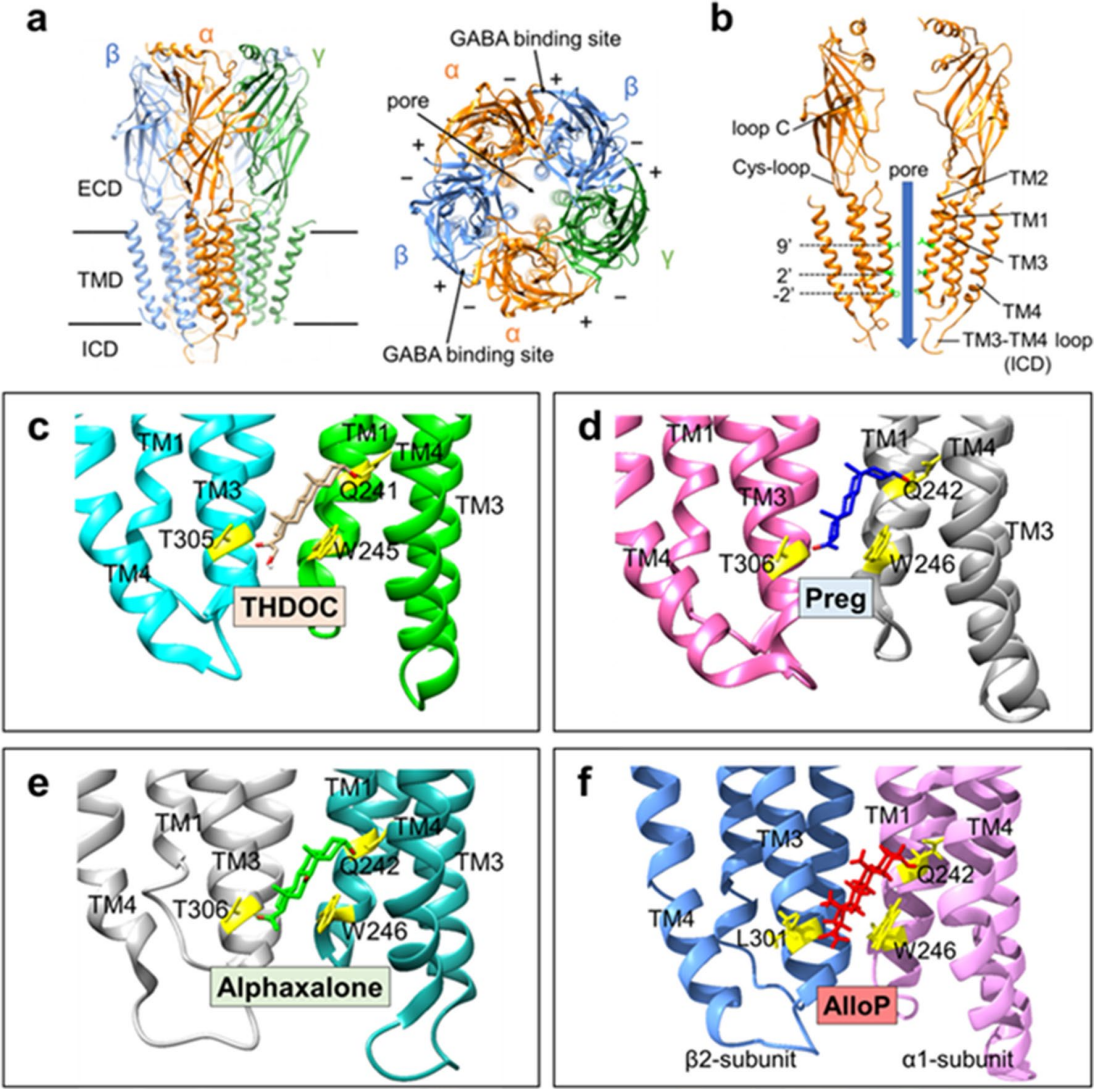
Model structures of GABA_A_ receptor and neurosteroid binding sites. (**a**) Cryogenic-EM structure showing the side view and top view of GABA_A_ receptor α1β3γ2 (PDB: 6I53). (**b**) α-subunits of GABA_A_ receptor α1β3γ2 (PDB: 6I53) and pore. (**c**–**e**) X-ray crystallographic structures of GABA_A_ receptor TMD intersubunit site with ligand. (**c**) GLIC-GABA_A_ receptor—THDOC (PDB: 5OSB). (**d**) β3 ECD-α5 TMD GABA_A_ receptor—Preg (PDB: 5O8F). (**e**) ELIC-GABA_A_ receptor—alphaxalone (PDB: 6CDU). (**f**) GABA_A_ receptor *α*1*β*2*γ*2—AlloP (PDB: 8SI9)

GABA_A_ receptors are the primary mediators of inhibitory neurotransmission in the CNS. They are members of the pentameric ligand-gated ion channel (pLGIC) superfamily, which includes nicotinic acetylcholine receptors, serotonin receptors, and glycine receptors. pLGICs consist of five subunits arranged around a central ion-conducting pore that increases its permeability to certain ions following neurotransmitter binding [46]. GABA_A_ receptors are constituted from a family of 19 homologous subunit genes (*α*1–6, *β1*–3, *γ*1–3, *δ*, *ε*, *θ*, *π*, and *σ*1–3) [47, 48]. Although there are thousands of possible combinations of GABA_A_ subunits, very few have been identified in situ [47]. GABA_A_ receptors are usually composed of two α subunits, two copies of a single β subunit, and one additional subunit, such as *γ*, *δ*, or *ε* [47, 48] (Fig. 3a). The most abundant combination of subunits in the mammalian brain is *α*1*β*2*γ*2, which is estimated to account for 60% of all GABA_A_ receptors [49, 50]. GABA_A_ receptor isoforms differ in their regional expression throughout the nervous system, developmental expression, and even subcellular localization [51].

Each GABA_A_ receptor subunit is composed of a large extracellular domain (ECD), a transmembrane domain (TMD) formed by four membrane-spanning helices (TM1–4), an intracellular domain (ICD) which contains a long intracellular loop between TM3 and TM4, and a short extracellular C terminus (Fig. 3a). Five subunits assemble in a circle around a pore formed by the TM2’s of each subunit, which functions as a chloride-selective ion channel. Binding sites for the activating ligand, GABA, are located at the interface between the ECDs of the *β* and *α* subunits [52, 53] (Fig. 3a). The portion of the GABA binding site containing the defined loops ‘A, B, and C’ is on the *β* subunit (designated as the ‘+’ or ‘principle’ subunit), while the loops ‘D, E, and F’ are on the *α* subunit (designated as the ‘−’ or ‘complementary’ subunit); the GABA binding site is described as the ‘*β*+/*α*−’ interface [47]. Interestingly, histamine selectively binds to the *β*–*β* interface acting as an agonist in *β*-homomeric receptors [54, 55] and extra-synaptic receptors in which there is a *β*–*β* interface [56]. Hence, GABA_A_ receptors composed of various subunit combinations contain subunit interfaces that constitute distinct binding sites for GABA and histamine.

### (2) Modulation of GABA_A_ receptors

Presynaptically released GABA diffuses across the synaptic cleft and activates post-synaptic GABA_A_ receptors. GABA binding leads to a series of conformational changes in GABA_A_ receptors [57, 58]. First, GABA binding causes the closure of the *β*+/*α*− (binding site) interface and an anti-clockwise asymmetric rotation of all subunit ECDs, followed by a clockwise rotation of the TMD. This TMD movement tilts the TM2 helices, rotating the 9’ Leucine (this 9’ residue composes the gate responsible for opening and closing the pore, Fig. 3b) away from the pore. These conformational changes bring about pore opening and permit chloride ion (Cl^−^) flux. Notably, the direction of Cl^−^ flow depends on the electrochemical gradient of Cl^−^ [59, 60]. Immature neurons express high levels of Na–K–2Cl cotransporter isoform 1 (NKCC1) which mediates Cl^−^ uptake and maintains high intracellular Cl^−^ concentration. Therefore, GABA_A_ receptor opening causes an efflux of Cl^−^ and mediates depolarization. In mature neurons, NKCC1 is downregulated and replaced with K–Cl cotransporter isoform 2 (KCC2) [59, 60], which exports Cl^−^ and keeps the intracellular Cl^−^ concentration low. Thus, GABA_A_ receptor opening leads to Cl^−^ influx and causes hyperpolarization. This hyperpolarization prevents action potential generation by shunting the depolarization produced by excitatory neurotransmission.

*αβγ*-GABA_A_ receptors are preferentially expressed in post-synaptic membranes. When GABA is released presynaptically, it rapidly reaches a high concentration in the synapse, maximally activating GABA_A_ receptors. The concentration of GABA in the synaptic cleft decreases rapidly, due to binding to the receptor and diffusion and reuptake by membrane transporters [26, 61]. The synaptic current mediated by GABA_A_ receptor rapidly decays due to the dual processes of desensitization (channel closing with GABA occupancy) and deactivation (channel closure due to GABA unbinding). This large, short-lived synaptic current is referred to as a phasic inhibitory current. Recent structural studies have provided some details regarding mechanisms of desensitization [57, 58, 62, 63]. In the desensitized state, unlike the resting or open state, the intracellular end of the pore (2ʹ and −2ʹ residues, Fig. 2b) is closed, preventing chloride flux. Desensitization is a negative feedback mechanism that prevents receptor overactivation in pathological states and decreases postsynaptic currents with consecutive synaptic neurotransmitter release [64, 65].

There are also extra-synaptic GABA_A_ receptors located outside of the synapse on the neuronal plasma membrane, where they are exposed to low levels of GABA which leaks from the synapse [66]. Extra-synaptic receptors provide a persistent (non-desensitizing) conductance. These persistent currents are called tonic inhibitory currents and produce membrane hyperpolarization that serves to control neuronal excitability [66, 67]. Most extra-synaptic GABA_A_ receptors contain *δ* subunits (rather than *γ* subunits) [66, 68], together with *β*2 or *β*3 and specific *α* subunit isoforms (often *α*4, *α*5, or *α*6). These combinations of subunits exhibit a high affinity to GABA and are activated with low concentrations of GABA [67, 69, 70]. These receptors are predominantly expressed in the hippocampus, thalamus, and cerebellum [70, 71]. GABA_A_ receptors containing the *α*4 subunit are expressed in dentate gyrus granule cells and thalamic neurons, whereas receptors containing the *α*6 subunit present ubiquitously in cerebellar granule cells. In addition, *α*5 combines with *γ* subunits rather than *δ* in CA1/3 pyramidal cells [72–74].

### (3) Neurosteroid modulation of GABA_A_ receptors

NS such as AlloP, Preg, and THDOC activate GABA_A_ receptors and are referred to as PAM-NS. At low concentrations, PAM-NS enhance the effect of GABA on GABA_A_ receptor currents (referred to as potentiation) while at higher concentrations they can elicit GABA_A_ currents in the absence of GABA (referred to as direct activation) [26–28]. Conversely, PS, DHEAS, and Epi-AlloP inhibit GABA_A_ receptors and are referred to as NAM-NS [30, 31, 75].

Studies over the past twenty years have begun to reveal the mechanisms through which NS modulate GABA_A_ receptor function. It has become clear that NS potentiation and direct activation of GABA_A_ receptors are both the result of specific NS binding interactions with sites in the TMDs of the receptor protein [76–78]. This was initially demonstrated by site-directed mutagenesis studies in which the effect of amino acid substitutions in the TMDs of *α*_1_*β*_2_*γ*_2_ GABA_A_ receptors on NS-mediated enhancement of GABA_A_ currents was assessed [76, 79]. These studies identified several residues in the *α*_1_ and *β*_2_ subunits that were critical for NS action. Notably, mutations of residue Q241 (refers to the amino acid type of the residue and its number in the protein sequence) in TM1 of the *α*_1_ subunit markedly reduced the effects of PAM-NS. Subsequent X-ray crystallographic studies of homopentameric chimeric receptors in which the TMDs are all from *α*-subunits have shown that PAM-NS (Preg, THDOC, alphaxalone) bind between adjacent subunits forming a critical hydrogen bond between the 3*α*-OH group of the NS and residue Q241 (or 242) [80–82] (Fig. 3c–e). Recent Cryo-EM studies have demonstrated that in *α*_1_*β*_2_*γ*_2_ receptors [83], the PAM-NS, AlloP, selectively binds in the interface between the *β*_2_(+) and *α*_1_(−) subunits, forming a hydrogen bond with residue Q242 (Fig. 3f). Another cryo-EM study has demonstrated that the sulfated-NAM-NS, PS, has a unique site of action, binding within the ion channel pore, where it acts to block ion flux [83].

Photoaffinity labeling studies also show that PAM-NS bind in the interface between the *β*_3_(+) and *α*_1_(−) subunits [28, 84] and demonstrate that the enantioselectivity of AlloP results from differential binding to this site [85]. Photolabeling has identified additional intrasubunit NS binding sites contained within the TMDs of the *α*_1_ and *β*_3_ subunits [28]. Mutagenesis of residues in the *α*_1_-intrasubunit binding site shows that AlloP binding to this site potentiates GABA-elicited currents, albeit with less efficacy than binding to the *β*_3_(+)/*α*_1_(−) intersubunit site [86]. In contrast, AlloP binding to the *β*_3_-intrasubunit site inhibits GABA-elicited currents by promoting receptor desensitization [31]. Interestingly the 3β-OH NAM-NS, such as Epi-AlloP bind in the *α*_1_- and *β*_3_-intrasubunit sites but not in the β_3_( +)/α_1_(−) intersubunit site [31]. Epi-AlloP binding to either the *α*_1_- or *β*_3_-intrasubunit sites promotes desensitization, explaining its action as a NAM-NS. Collectively, these data demonstrate that there are multiple NS binding sites on the GABA_A_ receptor, with overlapping subsets of sites for the 3α-OH and 3β-OH NS and a unique pore blocking site for the sulfated NS. These data are consistent with earlier findings that 3β-OH and sulfated NAM-NS are non-competitive inhibitors of PAM-NS action and that 3β-OH and sulfated NAM-NS act at distinct sites [26, 87]. These structural findings illustrate that small changes in NS structure can alter both the sites at which NS act and their functional effect on GABA_A_ receptors, providing an opportunity for development of NS drugs with increased specificity.

## Prospects for a neurosteroid anesthetic

While NS bind to different sites on the GABA_A_ receptor than etomidate, propofol or barbiturates, they share a common mechanism of anesthetic action with these drugs, acting as positive allosteric modulators of GABA_A_ function [88–91]. However, the side effect profile of NS is markedly different from the other intravenous anesthetics, suggesting major differences in off-target binding and or effect. For example, anesthetic doses of propofol produce respiratory and cardiovascular depression [92, 93], and prolonged high-dose infusion causes propofol infusion syndrome, a rare but fatal condition [94]. Although etomidate minimally perturbs cardiorespiratory function, it has a distinct off-target profile with adrenocortical suppression resulting from inhibition of the cytochrome P450 enzyme, 11β-hydroxylase [95, 96]. In contrast, NS elicit minimal cardiorespiratory depression and have beneficial off-target effects such as neuroprotective and anti-inflammatory actions [9, 97]. The beneficial effects of NS, coupled with minimal adverse effects, suggest that a NS scaffold provides an excellent basis for developing new anesthetics.

### (1) Prevention of neurological complications

Peri-operative neurocognitive disorder (PND), traditionally described as post-operative delirium (POD) and postoperative cognitive dysfunction (POCD), is associated with increased post-operative complications, prolonged hospital stay, decreased quality of life and higher mortality [98–100]. Although the pathogenesis of PND remains unknown, neuroinflammation caused by surgical trauma or pain and manifest as over-activation of microglia is a proposed mechanism [101–103]. While it is unclear whether commonly used anesthetics directly cause or exacerbate neuroinflammation [104–107], there is substantial evidence that NS attenuate neuroinflammation. Indeed, NS are being developed as treatments for a variety of neurodegenerative disorders, based on their ability to regulate neuroinflammation (Table 1).

One mechanism through which NS might inhibit neuroinflammation is by activating microglial GABA_A_ receptors and inhibiting inflammatory signaling [108]. However, other anesthetics also activate GABA_A_ receptors and are not anti-inflammatory, suggesting different or additional targets. Indeed, ent-AlloP, which has minimal effect on GABA_A_ currents, has been reported to attenuate neuroinflammation as effectively as AlloP [109]. There are several non-GABAergic molecular pathways through which neurosteroids have been shown to modulate neuroinflammation.

One pathway through which NS inhibit neuroinflammation is by stimulating the secretion of brain-derived neurotrophic factor (BDNF) [110, 111]. BDNF is a trophic factor involved in neuronal survival, regeneration, and synaptic function through its receptor, TrkB (129). BDNF and TrkB are expressed in microglia, and their upregulation attenuates microglial activation [112]. Thus, the levels of BDNF and TrkB in microglia are negatively correlated with neuroinflammation [113]. Furthermore, BDNF and TrkB expression decrease with age, suggesting an explanation for the increased incidence of PND in geriatric patients [98–100]. In neurodegenerative disease and brain injury, BDNF has also been reported as an important factor for neuroprotection [111, 114, 115]. NS activation of pregnane X receptor (PXR) has been suggested as a mechanism for increased BDNF secretion. PXR is an orphan nuclear receptor that is activated by pregnane and PXR activation enhances BDNF secretion [116]. AlloP and alphaxalone have been shown to both activate PXR and exert neuroprotective effects [109, 117]. Notably, in a human RCT comparing Phaxan, propofol, and sevoflurane in hip replacement surgery [118], patients in the Phaxan group exhibited better postoperative cognition accompanied by higher levels of plasma BDNF compared with propofol- and sevoflurane-treated patients.

NS can also inhibit neuroinflammation by stimulating autophagy. Autophagy is a process through which cells degrade and recycle cellular components and dysfunction of autophagy is associated with microglial activation and neurodegeneration [119–121]. AlloP has been shown to reduce neuroinflammation and neurodegeneration by activating autophagy [122]. Recent studies also indicate that NS can inhibit the innate immune system and microglial activation by inhibiting toll-like receptors (TLR) signaling [123]. TLRs are expressed in sentinel cells, including microglia, and are activated by pathogen-associated molecular patterns leading to the production of proinflammatory cytokines. Over-activation of TLRs has been associated with both depression and neurodegeneration [124, 125]. A recent study reported that surgical trauma activates TLRs, leading to neuroinflammation and PND [126]. While the precise mechanism(s) through which NS inhibit microglial activation and neuroinflammation is an active subject of investigation, the ability of NS to inhibit neuroinflammation indicates that a NS anesthetic may reduce the incidence and severity of PND observed with current anesthetics.

Anesthetic neurotoxicity in infants is also a serious clinical issue. While two large human studies [127, 128] have shown that a short duration of general anesthesia in infants and young children does not cause apparent developmental or persistent behavioral effects, there is substantial evidence that anesthetics cause neurotoxicity in the developing animal brain, leading to long-term learning and behavioral deficits [129–131]. Thus, concern remains that prolonged or repeated exposure to anesthetics may produce long-term behavioral effects in infants. Unlike other anesthetics, NS are not associated with neurotoxicity when administered to neonatal animals. In a study comparing the effects of NS and propofol anesthesia in neonatal mice, propofol produced significant neuronal apoptosis, whereas alphaxalone did not (9). Alphaxolone has also been reported to attenuate the neuronal injury caused by isoflurane administration in fetal and neonatal rats [132]. It is postulated that anesthetic GABA_A_ receptor activation and/or NMDA receptor inhibition causes neuronal apoptosis [130, 133]. Since propofol and PAM-NS both produce anesthesia by enhancing GABA_A_ receptors, their divergent effects on neonatal neurotoxicity are likely mediated by an off-target neuroprotective effect of NS. In this regard, there may be overlapping mechanisms between the protective effects of NS in the developing brain and the anti-neuroinflammatory effects of NS discussed above [134, 135]. An alternative explanation for the divergent neuroprotective effects of NS and propofol in neonatal animals has also been postulated. In their study comparing alphaxalone and propofol, Tesic et. al. observed that alphaxalone, but not propofol, reduced presynaptic GABA release [9]. They hypothesized that NS may thus prevent excessive activation of GABA_A_ receptors and prevent neurotoxicity. While it remains mechanistically unclear how NS protect the neonatal and adult brain from injury, their neuroprotective effects provide a strong basis for development and use of a NS anesthetic.

### (2) Anesthetic antagonists and partial agonists

An agent that antagonizes NS action could be useful as a reversal agent for a NS anesthetic and as a research probe to understand the effects of endogenous NS on behavior and mood. NS have more complex structures than propofol or barbiturates, allowing synthesis of a multitude of analogues each assuming a unique pose in its GABA_A_ receptor binding site. Therefore, it may be possible to design analogues that are either general NS antagonists, or that bind to and/or act at specific NS binding sites on GABA_A_ receptors. One such compound, (3α,5α)-17-phenylandrost-16-en-3-ol (17PA), has been synthesized and tested [136]. 17-PA antagonizes the positive allosteric effects of AlloP and other 3α5α-OH PAM-NS as well as their anesthetic actions in tadpoles and rats [137]. The antagonist actions of 17-PA are NS specific and do not affect barbiturate or benzodiazepine action. 17-PA is, however, a low-affinity antagonist and shows a perplexing preferential antagonism of 3α5α-OH vs 3α5β-OH NS. Nonetheless, it demonstrates the feasibility of developing an anesthetic NS antagonist.

Another area of clinical need is for agents that can produce a maximal level of sedation or depth of anesthesia, regardless of the dose infused. This type of agent would be particularly useful for safely providing moderate or deep sedation without risk of inadvertent general anesthesia or overdose. In principle, this could be accomplished using an anesthetic analogue that maximally increases GABAergic currents to a lesser extent than the parent compound; this is referred to as a partial agonist. There are numerous examples of NS that are partial GABA_A_ receptor agonists. For example, various pregnanediols can enhance GABAergic currents to varying degrees [138]. The partial agonist effects of NS may be explained either by varying efficacy, or selective action at a subset of NS binding sites. NS present a particularly attractive for developing partial agonists because of their favorable off-target profile and because of the existence of multiple functional binding sites on the GABA_A_ receptor.

## Conclusion

NS bind to specific sites on GABA_A_ receptors, thus modulating inhibitory signaling in the nervous system. Endogenous NS mediate changes in mood and behavior, whereas exogenously administered NS act as general anesthetics. NS have a favorable profile of off-target effects compared to other parenteral anesthetics, causing minimal cardiovascular or respiratory depression while providing anti-inflammatory and neuroprotective actions. This favorable adverse effect profile renders NS an excellent scaffold for development of anesthetic agents to fill unmet clinical needs. Multiple functional binding sites for NS have been identified on the GABA_A_ receptor. Developing site-specific NS and/or NS varying in efficacy is an attractive approach for developing agents that can produce deep sedation without anesthesia. Development of a high affinity NS with minimal efficacy as a GABA_A_ receptor PAM is also an avenue for developing an antagonist (reversal agent) for NS-anesthetics. Given all of these factors, NS provide a promising scaffold for the development of anesthetics with improved safety and efficacy.
